# Acquisition of Cisplatin Resistance Shifts Head and Neck Squamous Cell Carcinoma Metabolism toward Neutralization of Oxidative Stress

**DOI:** 10.3390/cancers12061670

**Published:** 2020-06-24

**Authors:** Wangjie Yu, Yunyun Chen, Nagireddy Putluri, Cristian Coarfa, Matthew J. Robertson, Vasanta Putluri, Fabio Stossi, Julien Dubrulle, Michael A. Mancini, Jonathan C. Pang, Trung Nguyen, Dodge Baluya, Jeffrey N. Myers, Stephen Y. Lai, Vlad C. Sandulache

**Affiliations:** 1 Bobby R. Alford Department of Otolaryngology Head and Neck Surgery, Baylor College of Medicine, Houston, TX 77030, USA; Wangjie.Yu@bcm.edu (W.Y.); jcp9@rice.edu (J.C.P.); trung97@nmsu.edu (T.N.); 2 Department of Head and Neck Surgery, University of Texas MD Anderson Cancer Center, Houston, TX 77030, USA; yunchen@mdanderson.org (Y.C.); jmyers@mdanderson.org (J.N.M.); SYLai@mdanderson.org (S.Y.L.); 3 Department of Molecular and Cellular Biology, Baylor College of Medicine, Houston, TX 77030, USA; putluri@bcm.edu (N.P.); coarfa@bcm.edu (C.C.); stossi@bcm.edu (F.S.); jdubrull@fredhutch.org (J.D.); mancini@bcm.edu (M.A.M.); 4 Center for Precision Environmental Health, Baylor College of Medicine, Houston, TX 77030, USA; 5 Advanced Technology Core, Dan Duncan Cancer Center, Baylor College of Medicine, Houston, TX 77030, USA; Matthew.Robertson@bcm.edu (M.J.R.); vputluri@bcm.edu (V.P.); 6 GCC Center for Advanced Microscopy and Image Informatics, Houston, TX 77030, USA; 7 Chemical Imaging Research Center (CIRC), University of Texas MD Anderson Cancer Center, Houston, TX 77030, USA; DLBaluya@mdanderson.org

**Keywords:** cisplatin, head and neck cancer, fatty acid, ferroptosis, amino acid, oxidative stress

## Abstract

*Background*: Cisplatin (CDDP) is commonly utilized in the treatment of advanced solid tumors including head and neck squamous cell carcinoma (HNSCC). Cisplatin response remains highly variable among individual tumors and development of cisplatin resistance is common. We hypothesized that development of cisplatin resistance is partially driven by metabolic reprogramming. *Methods*: Using a pre-clinical HNSCC model and an integrated approach to steady state metabolomics, metabolic flux and gene expression data we characterized the interaction between cisplatin resistance and metabolic reprogramming. *Results*: Cisplatin toxicity in HNSCC was driven by generation of intra-cellular oxidative stress. This was validated by demonstrating that acquisition of cisplatin resistance generates cross-resistance to ferroptosis agonists despite the fact that cisplatin itself does not trigger ferroptosis. Acquisition of cisplatin resistance dysregulated the expression of genes involved in amino acid, fatty acid metabolism and central carbon catabolic pathways, enhanced glucose catabolism and serine synthesis. Acute cisplatin exposure increased intra-tumoral levels of S-methyl-5-thiadenosine (MTA) precursors and metabotoxins indicative of generalized oxidative stress. *Conclusions:* Acquisition of cisplatin resistance is linked to metabolic recovery from oxidative stress. Although this portends poor effectiveness for directed metabolic targeting, it supports the potential for biomarker development of cisplatin effectiveness using an integrated approach.

## 1. Background

Despite the introduction of targeted agents over the last 3 decades, platinum derivatives remain the mainstay systemic agents for a wide variety of common solid tumors including lung cancer, ovarian cancer, cervical cancer and head and neck cancer [1,2,3,4,5,6]. For head and neck squamous cell carcinoma (HNSCC), cisplatin (CDDP) has been repeatedly confirmed as the gold standard systemic agent [3] and will likely remain so for the near future. As such, it is critical to continue to improve our understanding of cisplatin anti-tumor effects, development of cisplatin resistance and identify mechanisms to detect and potentially overcome cisplatin insensitivity and/or resistance.

Over the last decade, we have shown that generation of oxidative stress either through application of ionizing radiation (IR) or platinum derivatives triggers acute, reversible metabolic changes in a variety of tumor models including HNSCC [7,8,9,10]. This has allowed us to develop a relatively quantitative, albeit indirect relationship between metabolic perturbations linked to carbon flux and generation of intra-cellular oxidative stress and DNA damage [7,8,9,10]. Initially, this relationship was limited to the most common metabolic reaction common to solid tumors, namely the conversion of pyruvate into lactate, the lynchpin of the Warburg effect [7,8,9,10]. More recently we showed that cisplatin effects on tumor metabolism propagate through central carbon metabolic pathways such as the pentose phosphate pathway, glycolysis and the Krebs cycle [11]. The relationship between cisplatin exposure and metabolic programming in tumor cells is important for two distinct yet overlapping reasons. First, metabolic pathways critical to cisplatin processing, if effectively targeted could generate novel chemo-sensitizing strategies that move beyond simplistic metabolic inhibition of glycolysis and mitochondrial respiration which have heretofore been the cornerstone of anti-metabolic investigation [12,13,14,15,16,17,18]. Second, identifying metabolic pathways key to cisplatin processing could allow us to develop potentially sensitive and specific biomarker signatures for relative cisplatin responsiveness, a clinical deliverable which has heretofore escaped characterization. 

Based on our previous studies, we hypothesized that acquisition of cisplatin resistance is partially driven by metabolic reprogramming designed to adapt to cisplatin-induced oxidative stress. In the current study we evaluated this hypothesis using a combination of in vitro and in vivo metabolomics approaches designed to evaluate both acute and chronic cisplatin generated metabolic stress. Our data indicate that both acute and chronic cisplatin exposure trigger broad-based metabolic reprogramming in HNSCC cells and tumors consistent with activation of secondary catabolic pathways designed to survive oxidative and energetic stress. These changes are partially effected through differential gene expression and suggest the potential to generate an accurate metabolomics signature of cisplatin effects in HNSCC.

## 2. Methods

The manuscript did not include any human data or experimentation. All animal experiments performed in the study were performed following approval of and in compliance with the Institutional Animal Care and Use Committee guidelines of the Baylor College of Medicine and the University of Texas MD Anderson Cancer Center (AN7291- 10/11/2016; 00000959-RN 02- 1/30/2018 respectively).

### 2.1. Cells

Cell lines (head and neck squamous cell carcinoma-HNSCC; FADU, PCI-13, HN30, HN31) were obtained from an established cell line bank in the laboratory of Dr. Jeffrey N. Myers under approved institutional protocols and authenticated using short tandem repeat analysis every 3 months [19]. Cisplatin (CDDP) resistant cells HN30R4 and HN30R8 were generated from HN30 by gradually increasing the cisplatin concentration in culture media from 0.3 μM to 4 μM and then to 8 μM respectively at a rate of approximately 1 μM every 2 weeks. Monoclonal populations, R4e1, R4b3, R4f5 and R4f6 were generated from HN30R4 pooled population by limiting dilution. HN30R4 and its clones were maintained in growth media containing 4 μM cisplatin and HN30R8 in 8 μM cisplatin for the remainder of the experimental period except when cisplatin was withdrawn for specific individual experiments. HN30R4 was generated at 8–9 weeks following initial cisplatin treatment. Establishment and propagation of HN30R4 clones was performed over an additional 3 months. HN30R8 was generated at ~21 weeks following initial cisplatin treatment. All data generated using HN30R4 and its derivative clones were obtained between 6 and 17 months following initial establishment; cells were maintained in growth media containing 4 μM cisplatin during that time period. Data using HN30R8 were obtained between 3 and 6 months following initial establishment; cells were maintained in growth media containing 8 μM cisplatin during that time period.

### 2.2. Drug Effect Studies

Drug effects were assayed using either clonogenic survival assays or using total DNA content as a surrogate for cell number [20]. For clonogenic assays, cells were treated with the indicated drug for 24 h then incubated for colony formation for 10–14 days, fixed and stained (0.05% crystal violet in 10% formalin solution). Colony surviving fractions were determined based upon the plating efficiency of the control group. For drug effect measurements cells were seeded in 96-well plates and exposed to various drug concentrations. Drug effects were ascertained 72 h later using total DNA content as a surrogate for cell number [20]. Ferroptosis experiments were carried out using one of 3 ferroptosis agonists—RSL3, erastin (ERST) and ML210. All 3 agonists were used in the presence or absence of NAC [3 mM] or ferrostatin [1 μM] to evaluate the interaction between cisplatin sensitivity and ferroptosis sensitivity. For all cytotoxicity studies, cisplatin was removed from the growth media at the time of plating cells, for no more than 24 h prior to initiation of specific drug experiments. 

For routine experimental methods previous described by our group and others including senescence, Western blotting, platinum measurements [11] and gamma H2AX analysis [11,21] details are given in the Supplementary Materials and Methods section.

### 2.3. RNA Sequencing and Analysis

The HN30 parental cell line and 4 HN30R4 clones (R4b3, R4e1, R4f5, R4f6) were harvested under logarithmic growth conditions (50%–70% confluence) to generate 3 biological replicates (individual plates harvested at different time points) for each cell line. Total RNAs were isolated using Qiagen RNeasy Mini Kit (Qiagen Inc. Germantown, MD, USA) from cells in normal maintenance conditions with confluence between 50%–80%. RNAs were subjected to on-column DNase I digestion. Additional details regarding data analysis are included in Supplemental Materials and Methods [22,23,24,25,26,27,28,29]. Principal component analysis (PCA) was performed in the R-statistical analysis environment using the filtered, normalized and log2 transformed data as described in the Supplemental Materials and Methods. A cisplatin resistance gene signature was found by taking the genes with a Benjamini-Hochberg (BH) adjusted *p*-value (*q*-value) less than or equal to 0.05 and a fold change of at least 1.5 for each individual clone compared to the parental cell line and then finding the genes that were in common amongst these comparisons (i.e., consistently up- or down- regulated across all clones). Gene set enrichment analysis (GSEA) was performed on the log2 fold change ranked gene list of a differential gene analysis that used a contrast that compared the averaged gene expression of all the clones to the parental cell line against the entire MsigDB database (v6.2) with significance achieved at *q* < 0.25.

### 2.4. Metabolomic Profiling by Targeted MS

Steady state and flux metabolomic experiments were performed as previously described by our group [11] with additional experiment details included in Supplemental Materials and Methods. Steady state metabolomics profiling of HN30 and its cisplatin resistant clones was performed using the same cell lines profiled using RNAseq above. Once again, each cell line/clone was harvested under logarithmic growth conditions (50%–70% confluence) to generate 3 biological replicates (individual plates harvested at different time points) for each cell line. Samples were normalized to an internal standard depending on the method used during processing—L-creatine was used for the Luna method, with a coefficient of variation (CV) of 0.12, L-zeatine (CV of 0.11) was used in the Negative method and L-tryptophan (CV of 0.11) was used for the Positive method. After normalization samples were log2 transformed and then differential expression was determined. Two approaches were performed to identify differential metabolites; we used a parametric t-test followed by multiple hypothesis testing correction (using the Benjamini-Hochberg method) or a two-tier topological mapping approach. Differential metabolites identified using a parametric t-test were considered significant if they had a *q*-value of less than 0.25. A metabolite was considered related to the cisplatin resistance if it was statistically significant in all clones compared to the parental cell line and was consistently up-regulated or down-regulated across all three clones. We defined a parametric t-test signature as all the metabolites that met these criteria. We also relaxed the requirement of statistical significance to only two out of the three clones. We used the R-package TTMap to implement the two-tier topological mapping approach to cluster and identify differentially expressed metabolites [29]. In the TTMap package we used a *p*-value cutoff of 0.05, a fold change cutoff of two and the parental cells were used as controls with the cisplatin clones as the test groups. After clustering the data using TTMap and identifying the differential, a metabolite resistance signature based on the TTMap results was generated by taking only the common metabolites to all three clonal cell lines that were consistently up- or down- regulated across all three samples.

For^13^C flux experiments, HN30 and HN30R4e1 (50–60% confluence, logarithmic growth phase; *n* = 4 samples/cell line/time point) were exposed to 10 mM all labeled glucose for 3, 16, 48, 72 and 96 h. Distribution of ^13^C label among metabolites was measured (Supplementary Materials and Methods) and direct comparisons were made between HN30 and its resistant clone at each time point (*t*-test; *p*-value threshold or 0.05).

### 2.5. Metabolite and RNA-Seq Data Integration

Our metabolite signature generated from the parametric t-test or the metabolite signature generated using the TTMap algorithm was integrated with the gene signature derived from the common list of genes identified from the RNA-seq analysis that were statistically significant (*q*-values less than or equal to 0.05), had a linear fold change of at least 1.5 and were consistently up or down regulated when compared to the parental cell line in all the clones. We first mapped the identified metabolites to the genes associated with them using HMDB database. We then found the overlap of the metabolite associated genes with the significant list of genes identified from the RNA-seq analysis. This list of overlapping genes was then used in hypergeometric overrepresentation to identify pathways from the MsigDB database (v6.2) that were significantly altered. The resulting *p*-values were corrected for multiple hypothesis testing using the Benjamini-Hochberg method and a *q*-value of 0.05 was used to identify significantly altered pathways.

### 2.6. HNSCC Tumors

All animal experiments performed in the study were performed following approval of and in compliance with the Institutional Animal Care and Use Committee guidelines of the Baylor College of Medicine and the University of Texas MD Anderson Cancer Center. Details of the animal manipulation are provided in Supplemental Materials and Methods. For flank tumors, cells (2 × 10^6^/mouse) were subcutaneously injected into the left and right flank of each animal [9,14]. For the HN30 experiment, 4 control mice received no treatment following tumor inoculation; 7 animals received a single dose of cisplatin, administered at a dose of 5 mg/kg via intravenous injection, using pharmaceutical grade cisplatin (Intas Pharmaceuticals Limited, Pharmez, Ahmedabad-382213, India). Tumors were harvested at 1 h and 6 h following cisplatin administration. For the HN31 experiment, 6 control mice received no treatment; 12 mice received a single cisplatin injection of either 2 mg/kg or 5 mg/kg. Tumors were harvested at 1 h post cisplatin administration. 

### 2.7. Statistical Analysis

Statistical analyses for the RNAseq and metabolomics analyses are detailed in the specific sections above. For all other experiments, in vitro experiments were carried out at least in triplicate (for each condition) and were repeated to ensure reproducibility. All statistical analysis for in vitro and in vivo experiments was conducted using two-tailed, Student’s *t*-test analysis with a cutoff *p*-value of 0.05 to demonstrate statistical significance.

## 3. Results

### 3.1. Cisplatin Anti-Tumor Effects are Linked to Generation of Oxidative Stress

**Figure 1:**
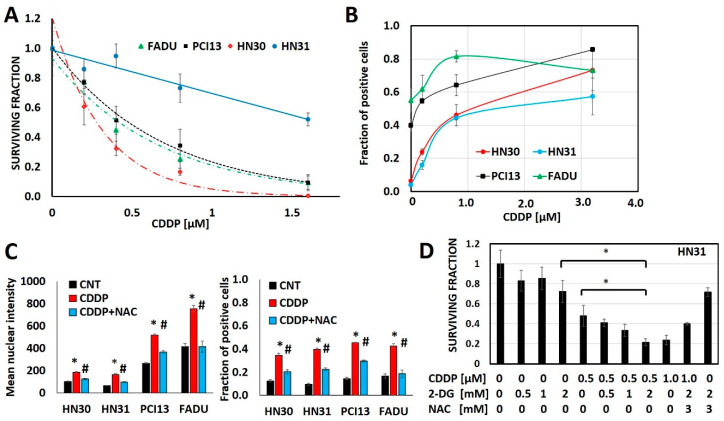
Cisplatin toxicity is a function of oxidative stress generation. (**A**) Head and neck squamous cell carcinoma (HNSCC) cells demonstrate differential cisplatin (CDDP) sensitivity as measured via surviving fraction in a clonogenic survival assay (CSA). (**B**) Cisplatin increases oxidative stress as measured via γH2ax fraction of positive cells in a dose dependent fashion across cell line backgrounds. (**C**,**D**) N-acetyl cysteine (NAC) reverses cisplatin induced increases in γH2ax (C; * denotes *p* < 0.05 for comparison of CDDP condition vs. control (CNT), # denotes *p* < 0.05 for comparison of CDDP+NAC vs. CDDP alone) and reverses cisplatin toxicity even when augmented by 2-deoxyglucose (2-DG). All data presented as averages, with error bars indicating standard error of the mean. * denotes *p* < 0.05.

Cisplatin generates oxidative stress, measured via consistent increases in γH2ax as a function of cisplatin concentration in 4 different HNSCC cell lines (HN30, HN31, FADU, PCI13) (Figure 1, Supplementary Figure S1). Glycolytic inhibitors that increase oxidative stress, like 2-deoxyglucose (2-DG) augment, while free radical scavengers like N-acetyl cysteine (NAC) reduce cisplatin’s anti-tumor cell effectiveness (Figure 1). 

**Figure 2:**
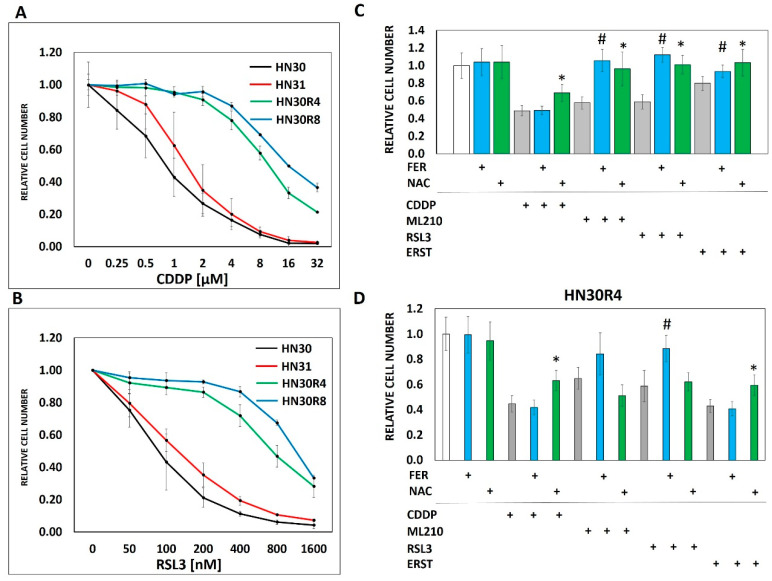
Conditioned cisplatin resistance generates cross-resistance to ferroptosis. (**A**) Exposure of HN30 cells to increasing concentrations of cisplatin (CDDP) resulted in generation of 2 resistant pooled populations (R4- cell line capable of proliferating in growth media containing 4 µM CDDP; R8- cell line capable of proliferating in growth media containing 8 µM CDDP). Both resistant pooled populations greatly surpassed the intrinsic cisplatin resistance demonstrated by HN31. (**B**) HN30R4 and HN30R8 demonstrated a significant decrease in sensitivity to RSL3 compared to the HN30 parental line. (**C**) Ferrostatin (FER) (1 μM) (blue bars) reversed the effects of RSL3, ERST and ML210 but not cisplatin on HN30 (72 h assay). NAC (3 mM) (green bars) reversed the effects of all 4 drugs (# indicates a statistically significant difference between drug alone and drug + ferrostatin using *t*-test, *p*-value <0.05; * indicates a statistically significant difference between drug alone and drug + NAC using t-test, *p*-value <0.05). (**D**) Sensitivity to both cisplatin and ferroptosis inducers was dramatically decreased in the HN30R4 pooled population compared to the parental HN30 cell line; ferrostatin (blue bars) and NAC reversal (green bars) of drug toxicity are partially abrogated (# indicates a statistically significant difference between drug alone and drug + ferrostatin using *t*-test, *p*-value <0.05; * indicates a statistically significant difference between drug alone and drug + NAC using *t*-test, *p*-value <0.05). Drug concentrations for HN30 are—CDDP 1µM, ML210 0.5 µM, RLS3 0.1 µM, ERST 2.0 µM. Drug concentrations for HN30R4 are—CDDP 16 µM, ML210 8 µM, RSL3 1.6 µM, ERST 16 µM. All data presented as averages, with error bars indicating standard error of the mean.

Cellular uptake of cisplatin is time and dose dependent with a linear relationship between the extracellular concentration and the intra-cellular concentration of cisplatin (Supplementary Figure S2). However, maintenance of intra-cellular cisplatin levels is at least partially energetically driven as demonstrated by a near 2-fold increase in both intra-cellular and DNA-bound cisplatin in response to glycolytic inhibition via 2-DG (Supplementary Figure S2). To evaluate the steady state interaction between cisplatin, oxidative stress and tumor metabolism we generated conditioned cisplatin resistance (>5-fold increase in IC_50_) in HN30, a wild-type *TP53* expressing, cisplatin sensitive HNSCC cell line (Figure 2A) [12,13,30]. Resistant cells (HN30R4) demonstrated decreased sensitivity to cisplatin at high micromolar concentrations and reduced reversal potential with NAC indicating saturation of anti-oxidant defense mechanisms (Figure 2). Cisplatin exposure increased global reactive oxygen species (ROS) levels in HN30 but not in HN30 R4e1, one of its resistant clones (Supplementary Figure S6); the increase in ROS levels was neutralized by NAC. Consistent with these results, cisplatin increased γH2ax in HN30R4 clones at much higher doses and to a lesser degree compared to the HN30 parental cell line and even its relative resistant HN31 counterpart which demonstrated saturation at lower doses (Supplementary Figure S5). Of note, conventional antioxidants such as 4-oxypiperidol and 6-hydroxy-2,5,7,8-tetramethylchroman-2-carboxylic acid generate significant single agent toxicity in our cells lines at micromolar and millimolar concentrations and thus cannot be used in the setting of cisplatin exposure.

### 3.2. Ferroptosis Cross-Resistance Indicates Reprogramming of the Oxidative Stress Response

Previous studies have shown that ferroptosis, a recently identified mode of programmed cell death mediated by oxidative stress and lipid peroxidation [31,32,33] may be triggered by cisplatin [34]. Based on these findings we speculated that acquisition of cisplatin resistance would result in decreased ferroptosis in resistant cells and clones in response to cisplatin exposure. Interestingly, cisplatin did not appear to trigger ferroptosis in our HNSCC model as evidenced by the inability of ferrostatin to reverse cisplatin toxicity in any of the tested cell lines (Figure 2C,D). However, acquisition of cisplatin resistance generated cross-resistance to ferroptosis inducers including erastin (cysteine-glutamate antiport system inhibitor), RSL3 and ML210, two chemically distinct inhibitors of glutathione peroxidase 4 (*GPX4*) (Figure 2B–D, Supplementary Figure S3). Unlike ferrostatin which was specific to reversing the effects of ferroptosis inducers, NAC reversed the anti-tumor cell activity of all 3 ferroptosis inducers as well as cisplatin (Figure 2, Supplementary Figure S3). The cross-resistance of HNSCC cells to both cisplatin and ferroptosis inducers despite the lack of direct cisplatin effects on ferroptosis prompted us to consider that both phenomena may be linked to cellular processing of oxidative stress. To test this hypothesis in more detail we generated individual clonal populations from the HN30R4 pooled cell population. All tested clones demonstrated consistent resistance to cisplatin yet maintained significant sensitivity to potentiation of cisplatin toxicity by 2-DG (Supplementary Figure S4).

### 3.3. Acquisition of Cisplatin Resistance in HNSCC is Accompanied by Dysregulation of Stress Response Pathways

**Figure 3:**
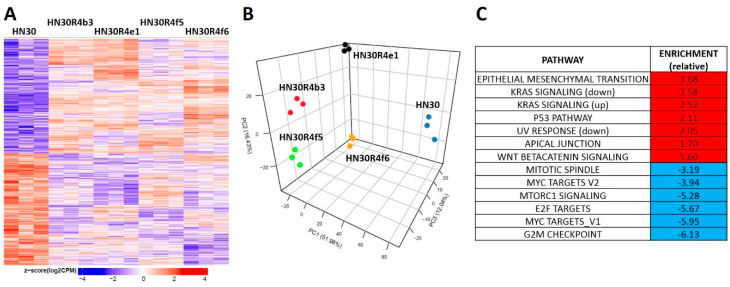
Differential gene expression patterns in cisplatin resistant HN30 clones. (**A**) Differential single gene expression differentiates the clones from the HN30 parental line (genes were considered statistically significant if they had a Benjamini-Hochberg (BH) adjusted *p*-value less than or equal 0.05 when the clones were compared to the parental line). (**B**) Principal component analysis differentiates the clones from the HN30 parental line. (**C**) HALLMARK pathways differentially enriched in the clones compared to the parental line.

HN30R4 derived clones demonstrated a gene expression signature distinct from that of the parental cell line (Figure 3A). Principal component analysis demonstrated that the clones have a unique gene expression profile compared to the parental cell line and that the acquisition of cisplatin resistance contributes the most to gene expression variability with clonal identity having a minor contribution to gene expression (Figure 3B). We identified 8046 genes that were statistical significant (*q*-value less than 0.05) for a contrast comparing the average of all four clones to the parental cell line. Of the significant genes 3968 (989 had a fold change of at least 1.5) were downregulated and 4078 were upregulated (2424 had a fold change of at least 1.5) (Figure 3A). Gene set enrichment analysis using previously defined Hallmark pathways demonstrated enrichment for genes involved in epithelial mesenchymal transition (EMT) and beta-catenin signaling along with depletion of genes associated with G2M checkpoint regulation in HN30 cisplatin resistant clones (Figure 3, Supplementary Table S1). HN30 maintains a wild-type *TP53* background and functional p53 activation in response to DNA damage [12,13,30]. To determine whether p53 signaling was preserved we tested for activation of p53 signaling (Supplementary Figure S5D). P53 stabilization and p21 activation was minimal in the R4 clones even at high cisplatin concentrations; however, resistant clones maintained the ability to undergo senescence, the primary mechanism of cell death in the HN30 cell line, consistent with a wild type *TP53* background. These effects were paralleled by enrichment of genes involved in the p53 response pathway (Figure 3C, Supplementary Table S1). Therefore, acquisition of cisplatin resistance generated gene expression shifts consistent with previously published literature and indicative of a stress response.

Since differential cisplatin transport has previously been shown to drive cisplatin resistance, we evaluated expression of individual genes in the cisplatin resistant clones. We detected modest changes in expression of ATP and ABC transporters (average expression in clones compared to parental line) but no differential expression of *ABCB1* (MDR1) (Supplementary Table S3). Functionally however, cellular cisplatin uptake was similar in the HN30 and HN30R4 lines, although DNA-bound cisplatin levels were significantly lower in the R4 line (Supplementary Figure S4B,C) but comparable with HN31 and disproportionately higher than would be consistent with the increase in relative IC_50_. Individual gene expression data provided a partial explanation for the cross-resistance to ferroptosis, namely upregulation of *SLC40A1*, a primary iron exporter (log fold change 7.2, *q*-value <0.001) and *GPX4* (log fold change 0.6, *q*-value <0.001) in resistant clones (Supplementary Table S2). 

### 3.4. Acquisition of Cisplatin Resistance Triggers an Enhanced Catabolic Phenotype Supported by Changes in Metabolic Gene Expression

**Figure 4:**
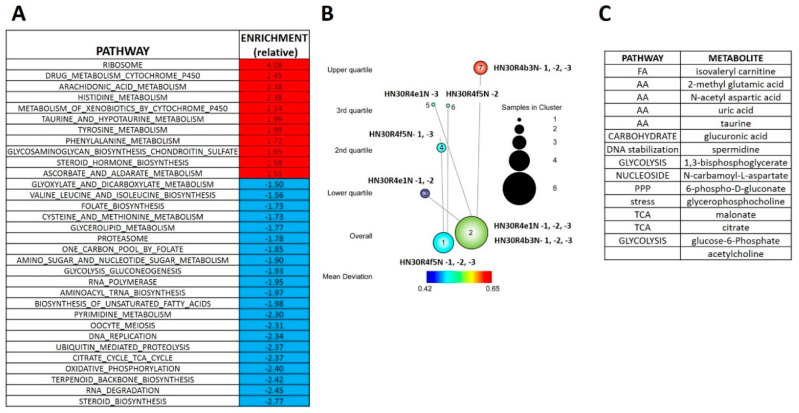
Differential metabolomics profile of cisplatin resistant HN30 clones. (**A**) Differential metabolic pathways enrichment differentiates the clones from the HN30 parental line. (**B**) Two tier topology mapping of the metabolite data identifies a set of metabolites which are different between the clones and the HN30 parental line (**C**).

**Figure 5:**
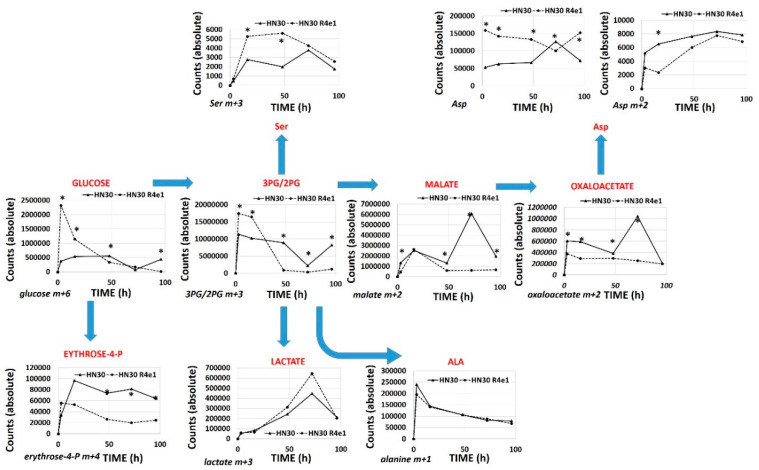
Differential ^13^C flux in cisplatin resistant cell lines. HN30 and HN30R4e1 were exposed to 10 mM all carbon labeled glucose (^13^C) for 3, 16, 48, 72 and 96 h (h) in the absence of unlabeled glucose. Incorporation of ^13^C label was measured into the above metabolites at each time point. Data are shown as average absolute counts (*n* = 4) with * indicating *p*-value <0.05 comparing HN30 R4e1 to HN30 for individual time points. Ala- alanine, Ser- serine, Asp- aspartate.

The RNAseq analysis reveals that cisplatin resistant clones demonstrated an enrichment of ribosomal and amino acid metabolism genes and depletion of TCA cycle, oxidative phosphorylation and fatty acid biosynthetic genes (Supplementary Table S4). Since differential metabolic gene expression does not necessarily translate into differential activity, we performed steady state unbiased metabolomics analysis (Supplementary Tables S5 and S6) and combined the gene expression and metabolomics data into an integrated dataset. We identified differentially expressed metabolites using either parametric t-tests or topology-based clustering (Figure 4B). A metabolite was considered deregulated by cisplatin if present in all three clones (Supplemental Table S5) or if present in two clones (Supplemental Table S6) with a statistically significant change in the metabolite compared to the parental cell line in the same direction, either increasing or decreasing. Clustering revealed two global clusters with two clones (HN30R4e1 and HN30R4b3) with a similar deviation in metabolite expression compared to the parental cell line than the third (HN30R4f5). Local clustering in the overall cluster two showed that one clone (R4ein) had a larger variation in metabolite expression than the other (HN30R4b3) (Figure 4B). In the topology based clustering approach, the metabolites in common between the overall global clusters one and two that were statistically significantly changed with a similar trend in the expression change when compared to the controls (either decreasing or increasing) were considered deregulated by cisplatin (Figure 4C, Supplementary Table S7). An integrated approach of metabolomics and expression data identified cellular pathways differentially regulated in the cisplatin resistant clones (Supplementary Table S8; analysis limited to GO, KEGG and HALLMARK pathways). The primary significantly differentially regulated GO and KEGG pathways centered on small molecule, amino acid and fatty acid catabolism and anabolism (Supplementary Table S8). ^13^C flux experiments demonstrated higher glucose uptake and flux into 3-carbon intermediates and TCA cycle intermediates, with decreased incorporation into pentose phosphate pathway (PPP) intermediates in the cisplatin-resistant cells consistent with increased overall catabolic activity (Figure 5). The Warburg effect (conversion of glucose into lactate) was similar between parental and cisplatin resistant cells, consistent with our previous work [8,9,10,11]. However, cisplatin resistant cells demonstrated drastically different rates of serine and aspartate catabolism and synthesis (Figure 5).

### 3.5. Acute Cisplatin Exposure Generates Shifts Consistent with a Metabolic Stress Response

**Figure 6:**
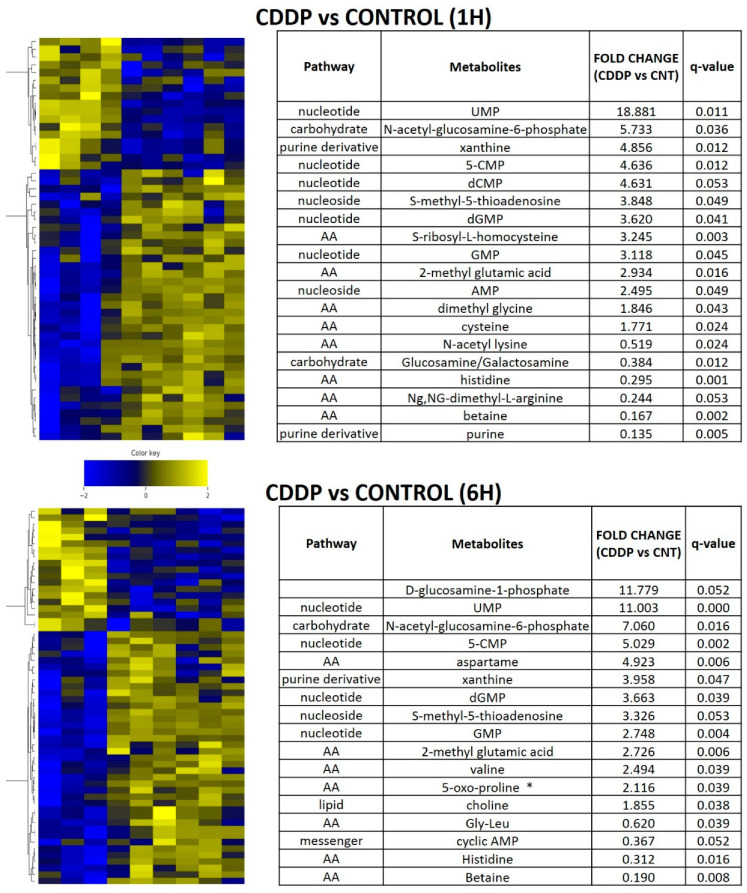
Acute metabolomic shifts secondary to cisplatin exposure. HN30 tumors were exposed to a single dose of cisplatin (CDDP) 5 mg/kg for 1 or 6 h (h); (control *n* = 4, CDDP *n* = 7). Heatmaps indicate metabolite levels which increased and/or decreased in CDDP treatment tumors compared to the control condition (FDR < 0.25). Tables summarize metabolites which demonstrated a statistically significant increase or decrease compared to the control condition. * indicates metabotoxin; MTA- S-methyl-5-thioadenosine.

In order to determine whether the metabolic shifts identified in the setting of chronic cisplatin exposure and resistance could be detected in the acute exposure setting, we performed in vivo experiments using the parental HN30 cell line (Figure 6). Cisplatin triggered a rapid shift in amino acid and fatty acid intermediates (consistent with in vitro data), along with accumulation of metabotoxins such as pyroglutamic acid. We detected diffuse alterations in the intrinsic cellular processing capability for methyl group transfer as demonstrated by significant decreases in intra-tumoral betaine levels, with increases in cysteine, dimethyl glycine and S-methyl-5-thiadenosine (MTA) (Figure 6). Consistent with these findings, steady state levels of homocysteine, choline, cystathionine, glutathione were consistently elevated compared to the parental HN30 line with decreased MTA and serine levels (consistent with the ^13^C flux data). HN31 tumors, (mutant *TP53* counterpart of HN30) demonstrated consistent alterations in glutathione and pyroglutamic acid along with differential levels of carnitine derivatives and 1,3 diphosphateglycerate (Supplementary Table S9) following single dose cisplatin administration (2 mg/kg or 5 mg/kg) at 1 h post administration (*n* = 6/condition). These results were consistent with the integrated analysis (Supplementary Table S8) which identified genes (i.e., *GAMT*) and pathways involved in methyl group transfer activity as differential regulated between the resistant clones and parental cell line.

## 4. Discussion

Response to cisplatin containing chemotherapy regimens is critical to maximizing oncologic control for HNSCC and other solid tumors [3,35]. Conversely, development of cisplatin resistance is a significant driver of tumor recurrence and disease-specific mortality. Although many mechanisms have been described which contribute to cisplatin response and development of resistance [36,37,38,39,40,41], little is known about what literally fuels this process within tumor cells. We previously showed that cisplatin generates transient changes in cellular reducing equivalents which correlate with relative HNSCC cell and tumor response [11,12]. More recently we have shown that these changes reverberate to common carbon pathways within HNSCC cells, including the conversion of pyruvate into lactate as well as transient shifts in other glycolytic, TCA and PPP intermediates [11]. In the current study we sought to extend this previous analysis in order to generate a more comprehensive picture of metabolic changes occurring in HNSCC in response to cisplatin exposure.

For this analysis we developed a secondary tool, namely HNSCC cells which are resistant to cisplatin through conditioned resistance. This tool allows us to study a critical steady state condition which was not previously available, since cisplatin exposure will eventually cause death in parental HNSCC cells within a relatively narrow IC_50_ range. Since the resistant cells are able to survive and proliferate in the presence of cisplatin, they remove the death condition and allow us to better delineate metabolic perturbations. We first used gene expression data to internally and externally validate our model. Shifts in *RAS* related genes are consistent with the mutant *HRAS* status present in the HN30/HN31 background. Enrichment of p53 related genes is consistent with persistent activation of wild type p53 activity measured in the resistant clones and the wild type *TP53* background of the parental cell line as is suppression of regulatory genes involved in the G2-M transition and persistence of senescence as the mechanism of cell death in HN30 cisplatin resistant clones. Finally, enrichment of EMT related genes correlates well with previously published literature on resistance to chemotherapy generally and cisplatin specifically [36,37,39,40,41]. Using both gene expression and functional assays, we showed that differential transport of cisplatin is likely not the primary driver of cisplatin resistance in this model, although secondary metabolic effects may partially impact transport. If the metabolic shifts identified here generated lower overall levels of ATP, this could impact relative intra-cellular cisplatin levels through energetically mediated differential import (i.e., copper transporter 1) and export (i.e., ATPase Cu2+ transporting beta polypeptide, MDR1) [37]. Furthermore, cisplatin resistance correlated with resistance to ferroptosis, despite the fact that cisplatin itself did not induce ferroptosis in our model, suggesting a broader shift in cellular processing of stress.

Based on our previous studies, we hypothesized that cisplatin exposure will shift HNSCC cell metabolism in a direction designed to ameliorate oxidative stress and allow for cell survival. The current dataset provides 4 lines of evidence supporting our hypothesis. First, the flux data clearly indicates rapid glucose uptake and catabolism to 3 carbon intermediates and flux into the TCA cycle in cisplatin resistant cells. Second, differential amino acid metabolism including serine and aspartate is consistent with a metabolic and oxidative stress condition. Serine biosynthesis from glucose via 3-carbon intermediates (i.e., 3-phosphoglycerate) has been linked to development of multiple tumor types [42,43,44] and differential expression of *PHGDH* appears to represent a component of the stress response under conditions of nutrient deprivation and/or oxidative stress [45,46,47,48]. Changes in aspartate consumption and generation are consistent with measurable shifts in oxaloacetate allowing for bidirectional carbon flux, which has now been shown to be driven by both acute cisplatin exposure and chronic exposure in the current study. These shifts under conditions of chronic exposure are supported by the transient shifts detected in response to cisplatin exposure in peptide/amino acid metabolism in vivo in HNSCC tumors. Third, cisplatin induced stress generates broad-based and significant shifts in both polyamine precursors and genes involved in this process. Polyamines such as putrescine, spermidine and spermine are critical to cell growth and survival including maintenance of protein and nucleic acid synthesis, stabilization of chromatin structure, differentiation, apoptosis and protection from oxidative damage. Polyamines are essential for normal cell growth and their depletion can result in cytostasis [49]. In cancer, polyamine metabolism is frequently dysregulated, overall indicating that elevated polyamine levels are necessary for transformation and tumor growth, consistent with the EMT and G2-M changes measured in response to chronic cisplatin exposure [50,51]. Fourth, the shifts in glutathione, pyroglutamic acid and MTA are indicative of a metabolic stress condition consistent with oxidative stress and DNA damage.

## 5. Conclusions

There are several translationally relevant conclusions which are supported by our current data. First, cisplatin exposure generates a metabolic signature which expands through common nodes into non central carbon processing pathways designed to support the stress condition. Diffuse distribution of metabolic stress across multiple overlapping pathways suggests that a single metabolic inhibitor is unlikely to generate profound cisplatin sensitization in this tumor type. This is consistent with the disappointing data generated using simplistic metabolic inhibition approaches targeting single metabolic pathways by us and others [12,13,14,30,52,53,54,55,56]. Second, acquisition of cisplatin resistance is associated with cross-over into other oxidative stress dependent pathways such as ferroptosis (to which Ras mutant cells are expected to be preferentially sensitive). This is despite the fact that cisplatin does not in-and-of-itself cause ferroptotic death and suggests reprogramming at a more global level. This phenomenon likely occurs through overlapping mechanisms including—1) increasing overall oxidative stress processing capacity which is critical for ferroptosis and 2) through more specific mechanisms such as differential processing of iron transport as seen through increased expression of *SLC11* (iron exporter). Third, metabolomics changes are at least partially reflected at a gene expression level. This is critical for translation. Since gene expression changes can now be ascertained using formalin fixed paraffin embedded tissue with high sensitivity and specificity, it becomes possible to interrogate metabolic reprogramming in patient derived specimens in the setting of clinical cisplatin utilization in order to generate an accurate signature of cisplatin response and conversely of cisplatin resistance. Finally, both acute and chronic cisplatin exposures are associated with generation of metabolites and metabotoxins which have significant potential for reprogramming of the tumor immune microenvironment such as histidine, which can regulate cytokine expression and pyroglutamic acid, which is associated with both inborn errors of metabolism and generalized metabolic stress (i.e., systemic acidosis) [57,58].

The current dataset is an important milestone in our translational efforts to build a metabolic signature of cisplatin response/resistance in HNSCC in order to support a precision oncology approach to its clinical utility. Using the pathways (genes and metabolites) identified here, we will expand our analysis to several other HNSCC cell lines under development in our laboratory and PDX models derived from patient tumors refractory to cisplatin resistant in order to generate a robust signature of cisplatin response. Since we now have validation that gene expression data can in fact reflect real metabolic shifts, we can use RNAseq and Nanostring technology to provide ultimate confirmation of the signature in archived human specimens as part of future translational efforts.

## Supplementary Materials

The following are available online at https://www.mdpi.com/2072-6694/12/6/1670/s1, Figure S1: Cisplatin increases γH2ax levels, Figure S2: Cisplatin uptake is dose and time dependent and partially energetically determined, Figure S3: Conditioned cisplatin resistance generates cross-resistance to ferroptosis, Figure S4: Cisplatin toxicity in cisplatin resistant HNSCC is responsive to metabolic inhibition and partially driven by DNA binding, Figure S5: Cisplatin maintains activity in resistant HNSCC cells, Figure S6: Cisplatin increases intra-cellular ROS levels, Table S1: Gene set enrichment in cisplatin resistant clones (Hallmark pathways), Table S2: Gene expression changes in cisplatin resistant clones, Table S3: Gene expression changes in cisplatin resistant clones (transporters and carriers), Table S4: Gene set enrichment in cisplatin resistant clones (KEGG pathways), Table S5: Differential steady state metabolite levels in cisplatin resistant clones (all 3 clones), Table S6: Differential steady state metabolite levels in cisplatin resistant clones (2 clones), Table S7: Correlation of dysregulated metabolites and metabolic genes in cisplatin resistant clones, Table S8: Integrated analysis of dysregulated metabolic pathways in cisplatin resistant clones, Table S9: Differential steady state metabolite levels following acute cisplatin exposure in HN31 tumors.
