# Supplementary material for: Acquisition of Cisplatin Resistance Shifts Head and Neck Squamous Cell Carcinoma Metabolism toward Neutralization of Oxidative Stress
Source: Cancers (Basel). 2020 Jun 24;12(6):1670. doi: 10.3390/cancers12061670 (PMC7352569; doi:10.3390/cancers12061670)
Supplement: Supplementary file 1 [file cancers-12-01670-s001.zip › Supplementary Files 6_2020/Supplementary methods and figures 6_2020.pdf]

Supplemental Materials and Methods

Chemicals and antibodies. N-acetyl cysteine (NAC), ferrostatin, erastin (ERST), RSL3, ML210 and cisplatin (CDDP) were purchased from Sigma-Aldrich (MO, USA). The following antibodies were used in this study: anti-p53 DO-1 (Santa Cruz Biotechnology, CA, USA), anti-phospho-P53 (Cell Signaling, MA, USA), anti-p21 (EMD Millipore, Billerica, MA, USA), p-histone gamma H2AX S139 (Cell signaling, MA, USA) and β-actin (Sigma, MO, USA).

Senescence. Senescence-associated (SA)-β-gal staining was carried out according to the manufacturer's instructions (Cell Signaling, MA, USA). Briefly, HNSCC cells plated at sub-confluence were exposed to various agents for 24hours. The media was then replaced with fresh media and cells were cultured normally for 5 days. Cells were then fixed for 10-15 minutes and stained overnight for SA-β-gal activity at 37°C. Blue-staining cells were scored as senescent and reported as a percentage of all the cells observed per high power field.

ROS measurements. Cells were incubated with various drugs for the time periods indicated for individual experiments (Figure legends, manuscript text). Media was then removed and cells were incubated with 2', 7' – dichlorodihydrofluorescein diacetate (H2DCFDA) [10µM] for 1 hour. Fluorescence measurements and imaging were performed using the Cytation 5 platform (Biotek, VT, USA).

Western blotting. Cells were lysated in radioimmunoprecipitation assay (RIPA) buffer (20 mM Tris at pH 7.5, 150 mM NaCl, 1 mM EDTA, 1 mM EGTA, 1% NP-40, 1% sodium deoxycholate) containing, 1 mM Na3VO4, 1 mM NaF, 1 mM β-glycerophosphate, 2.5 mM sodium pyrophosphate, and Roche Protease Inhibitor Cocktail. The lysate was sonicated and then centrifuged for 10 min. Supernatants were collected as protein extract. Concentrations of protein were determined by BCA Protein Assay (Thermo Scientific, IL, USA). Equal amounts of protein extract were loaded, transferred, and probed with indicated antibodies. Immunoblots were visualized using Clarity Western ECL (Bio-Rad, CA, USA).

Platinum measurements. Cells were exposed to cisplatin for varying periods of time in the presence or absence of other compounds. Platinum measurements were performed as recently described by our group using an induction coupled plasma – mass spectroscopy (ICP-MS) approach for both total intracellular and DNA-bound platinum.[11]

Gamma H2AX analysis. Cells were plated in 384 well glass bottom plates (Greiner Bio-One, NC, USA), treated with various drugs, fixed in 4% paraformaldehyde for 20 minutes on ice, quenched with ammonium chloride 0.1M in PBS for 15 minutes and permeabilized with PBS/Triton-X100 0.1% for 20 minutes at room temperature as previously described by our group.[11] Briefly, cells were incubated with anti phospho-γH2Ax rabbit polyclonal antibody at 1/500 (Cell Signaling Technology, MA, USA) followed by

Alexa488-conjugated anti-rabbit antibody (1/1000, Cell Signaling Technology, MA, USA) and counterstained with DAPI (2µg/ml). Plates were imaged on an IC200 (VALA Sciences, CA, USA) high-throughput microscope using a Nikon Plan Apo 20x/0.95 objective, 4 fields of view per well, one z-plane. Image analysis was performed using MyImageAnalysis, a web-based, PipelinePilot (Biovia)-powered image analysis application.[21] DAPI nuclear segmentation was used as a mask to extract mean pixel intensity values in the γH2Ax channel for each cell. Cells were considered γH2Ax positive when their mean pixel intensity was higher than the average pixel intensity plus one standard deviation of DMSO-treated cells. Between 35000 and 72000 cells were analyzed per condition. Graphs were generated in GraphPad Prism v.5.0.

Isotope labeling and profiling by targeted MS. Steady state and flux metabolomic experiments were performed as previously described by our group.[11] Nutrients labeled with ^{13}C were purchased from Cambridge Isotope Laboratories. Cells were grown in regular media until 60% confluence exposed to test conditions, followed by addition of 10mM D-glucose [U-$^{13}C_6$] glucose in growth media supplemented with 10%FBS, non-essential amino acids, glutamine and pyruvate. Cells from each treatment were snap-frozen with liquid nitrogen. Cells were scraped twice into a 0.5-ml mixture of 1:1 water/methanol, sonicated for 1 minute (two 30-second pulses), and then mixed with 450 µl ice-cold chloroform. The resulting homogenate was then mixed with 150 µl ice-cold water and vortexed again for 2 minutes. The homogenate was incubated at –20°C for 20 minutes and centrifuged at 4°C for 10 minutes to partition the aqueous and organic layers. The aqueous and organic layers were combined and dried at 37°C for 45 minutes in an automatic Environmental Speed Vac system (Thermo Fisher Scientific). The extract was reconstituted in a 500-µl solution of ice-cold methanol/water (1:1) and filtered through a 3-kDa molecular filter (Amicon Ultracel 3-kDa Membrane) at 4°C for 90 minutes to remove proteins. The filtrate was dried at 37°C for 45 minutes in a speed vacuum and stored at –80°C until MS analysis. Prior to MS analysis, the dried extract was resuspended in a 50-µl solution of methanol/water (1:1) containing 0.1% formic acid and then analyzed using multiple reaction monitoring (MRM). Ten microliters were injected and analyzed using a 6490 QQQ triple quadrupole mass spectrometer (Agilent Technologies) coupled to a 1290 Series HPLC system via selected reaction monitoring (SRM). Metabolites were targeted in both positive and negative ion modes: the electrospray source ionization (ESI) voltage was +4,000 V in positive ion mode and –3,500 V in negative ion mode. Approximately 9 to 12 data points were acquired per detected metabolite. To target the TCA flux, the samples were delivered to the mass spectrometer via normal-phase chromatography using a Luna Amino column (4 µm, 100A 2.1 × 150 mm). To target the fatty acid flux, the samples were delivered to the mass spectrometer via reverse-phase chromatography using a

Phenyl-Hexyl column (3 µm, 100A 2.1 × 150 mm). For ^{13}C-labeled experiments, SRM was performed for expected ^{13}C incorporation in various forms for targeted LC-MS/MS. Mass isotopomer distribution (MID) was calculated using the formula: [fractional incorporation = ($^{13}C/^{13}C + {}^{12}C$) × 100] and corrected for natural abundance. The change in reductive carboxylation flux was calculated by comparing the MIDs of TCA metabolites from [U-^{13}C]Glucose–labeled cells.

RNA sequencing and analysis. Total RNAs were isolated using Qiagen RNeasy Mini Kit from cells in normal maintenance conditions with confluence between 50% -80%. RNAs were subjected to on-column DNase I digestion. The Genomic and RNA Profiling Core first conducted Sample Quality checks using the NanoDrop spectrophotometer and Agilent Bioanalyzer 2100. We then used Illumina TruSeq Stranded mRNA library preparation protocol. Library prep kit: TruSeq Stranded mRNA (p/n 20020594), following user guide p/n 15031047, Rev E. A double-stranded DNA library was created using 250ng of total RNA (measured by picogreen), preparing the fragments for hybridization onto a flowcell. ERCC RNA Spike-In Controls were added to each sample according to the manufacturer's protocol. The resulting libraries were quantitated using the NanoDrop spectrophotometer and fragment size assessed with the Agilent Bioanalyzer. A qPCR quantitation was performed on the libraries to determine the concentration of adapter ligated fragments using Applied Biosystems ViiA7 Real-Time PCR System and a KAPA Library Quant Kit. The pooled library was loaded onto a NextSeq 500 High Output v2 flowcell (Illumina p/n FC-404-2005) and amplified by bridge amplification using the Illumina NextSeq 500 sequencing instrument. PhiX Control v3 adapter-ligated library was spiked-in at 1% by weight to ensure balanced diversity and to monitor clustering and sequencing performance. A paired-end 75 cycle run was used to sequence the flowcell on a NextSeq 500 Sequencing System.

RNA-seq Alignment, Quantification, and Analysis. Fastq files were trimmed using Trim Galore!.[22] After trimming reads were aligned to the human genome (GRCh38) using Hisat2.[23] Aligned reads were sorted using SAMtools and a summarized gene level count matrix of the sorted and aligned reads was generated using featureCounts. [24, 25] Differential gene expression analysis was performed using the EdgeR and limma packages within the R statistical analysis environment. Briefly, we filtered the data to remove low abundance genes and normalized the samples using the trimmed mean of M values (TMM) algorithm. Linear modeling using the limma package after implementing the voom method to account for the mean-variance relationship in the data was then performed to determine differentially expressed genes. [26, 27, 28]

HNSCC tumors. All animal experiments performed in the study were performed following approval of and in compliance with the Institutional Animal Care and Use Committee guidelines of the Baylor College of Medicine and the University of Texas MD Anderson Cancer Center. Female athymic nude mice (8-12

weeks) (Envigo, Indianapolis, IN) were maintained in a pathogen-free facility and fed irradiated mouse chow and autoclaved, reverse osmosis treated water. The animal facility was approved by the American Association for the Accreditation of Laboratory Animal Care and met all current regulations and standards of the U.S. Department of Agriculture, U.S. Department of Health and Human Services and the National Institutes of Health. All procedures were approved by the Institutional Animal Care and Use Committee of The University of Texas MD Anderson Cancer Center. For flank tumors, cells ($2x10^6$/mouse) were suspended in sterile phosphate buffered saline (Sigma, MO, USA) and injected (100µl volume for each injection) subcutaneously into the left and right flank of each animal using a 25-gauge needle. Animals were not anesthetized for this minimally invasive procedure. Tumor size was ascertained twice a week throughout the experimental period using manual measurements as previously described.[9, 14] Tumors were allowed to grow for 1 week prior to initiation of experiments; tumor growth did not exceed 1cm in the greatest dimension and did not result in skin ulceration in any animal. Animal weight was measured twice a week throughout the experimental time period. No weight loss was recorded for any of the animal experiments performed and no animal deaths occurred during the study period. The number of animals chosen for each *in vivo* experiment was based on previous experience with this animal model and the expected effect size. Statistical analysis of *in vivo* data was conducted as described below. For the animal experiment which generated HN30 tumors, 4 animals were used as controls and received no treatment following tumor inoculation; 7 animals received a single dose of cisplatin, administered at a dose of 5 mg/kg via intravenous injection, using pharmaceutical grade cisplatin (Intas Pharmaceuticals Limited, Pharmez, Ahmedabad-382213, India). One flank tumor from each animal was harvested at 1 hour following cisplatin administration. Briefly, each animal was anesthetized using an intramuscular ketamine/xylazine (75 mg/kg +5 mg/kg) mixture; an incision was made through the overlying skin using a sterile 15-blade scalpel and the tumor was removed using sterile technique. The skin edges were then re-approximated using sterile staples. Animals were monitored continuously until fully awake following the tumor harvest. The analgesic buprenorphine (0.10 mg/kg) was administered via intramuscular injection in the first hour post awakening from the procedure. At 6 hours following cisplatin administration the animals were euthanized using a CO2 chamber using the CO2/Euthanex standard procedure followed by cervical dislocation. The remaining flank tumor was then removed for analysis. For the experiment which generated HN31 tumors, the same protocol was utilized. The 6 control mice received no treatment; 12 mice received a single cisplatin injection of either 2mg/kg or 5mg/kg. Tumors were harvested at 1 hour post cisplatin administration following sacrifice of the animals using the above listed procedures. No

additional interventions were performed, and no additional special equipment was utilized for any of the listed animal procedures.

Supplementary Figure Legends

Supplementary Figure 1. Cisplatin increases γH2ax levels. Cisplatin generates a dose dependent increase in γH2ax mean nuclear intensity across cell line backgrounds. All data presented as averages, with error bars indicating standard error of the mean.

Supplementary Figure 2. Cisplatin uptake is dose and time dependent and partially energetically determined. A) HNSCC cells take up CDDP in a time and concentration dependent manner. Data presented as means with error bars indicating standard error of the mean. Inset tables indicate the relationship (R^2) between CDDP dose and intracellular platinum levels at each individual time point. B) Inhibition of glucose metabolism using 2-deoxyglucose (2-DG) (4hr pre-treatment) increases total intra-cellular platinum levels (3hr treatment) as well as DNA-bound platinum levels in HNSCC cells. All data presented as averages, with error bars indicating standard error of the mean. * indicates p-value <0.05 compared to control condition.

Supplementary Figure 3. Conditioned cisplatin resistance generates cross-resistance to ferroptosis. A, B) Exposure of HN30 cells to increasing concentrations of cisplatin resulted in generation of 2 resistant pooled populations (R4- cell line capable of proliferating in growth media containing 4µM CDDP; R8- cell line capable of proliferating in growth media containing 8µM CDDP). HN30R4 and HN30R8 demonstrated a significant decrease in sensitivity to erastin and ML210 compared to the HN30 parental line. All data presented as averages, with error bars indicating standard error of the mean. C) HNSCC cells were exposed to cisplatin (CDDP) - 1.5µM or RSL3 (RSL) - 40nM in the presence or absence of NAC- 3mM or ferrostatin (FER) - 1 µM for a total of 90 hours. Live cells were imaged every hour at the same location in each individual test condition. Representative images are provided for 0hr, 45hr and 90hr demonstrating drug effects on a specific cellular population.

Supplementary Figure 4. Cisplatin toxicity in cisplatin resistant HNSCC is responsive to metabolic inhibition and partially driven by DNA binding. A) Three distinct clones, isolated from the HN30R4 population were exposed to CDDP in the presence or absence of 2-DG. Relative drug effectiveness was ascertained using clonogenic survival assay. * indicates p<0.05 comparing the drug combination with either single drug condition. HN30: CDDP- 0.3µM, 2-DG- 2mM; HN30R4 clones: CDDP- 3.0µM, 2-DG- 2mM. B) Overall cellular platinum uptake by HN30, HN31 and HN30R4 cells was not significantly different (18hr; 10µM). C) DNA-bound platinum levels are lower in HN30R4 at 6 and 18hr post exposure (10µM) and in HN31 at 18hr post exposure compared to the HN30 cell line. * indicates p-value <0.05 compared to the HN30 cell line at the same time point.

Supplementary Figure 5. Cisplatin maintains activity in resistant HNSCC cells. Cisplatin (CDDP) exposure results in increased γH2ax positive cells (A) and mean nuclear intensity (B) in both sensitive and resistant cells; data are presented as means with error bars indicating standard error of the mean. Cisplatin triggers senescence measured via beta-galactosidase in both sensitive and resistant cells (C). * indicates p-value <0.05 for treated condition compared to control condition. Representative images are presented for each tested cell line. Resistant cells demonstrate reduced stabilization of p53 levels and activation of downstream p21 (D) compared to the sensitive parental HN30 line.

Supplementary Figure 6. Cisplatin increases intra-cellular ROS levels. A) Cells were plated in 96 well plates for 16 hours followed by treatment with CDDP [0, 5, 10µM] in the presence or absence of NAC [2mM]. ROS levels were measured via fluorescence using H2DCFDA. Data are presented as means, with error bars indicating standard error of the mean; * denotes $p < 0.05$ for individual comparisons indicated by the accompanying bars. B) Representative images of individual wells (control= no treatment; CDDP= 10µM cisplatin (16 hours), CDDP+NAC=10uM cisplatin + 2mM NAC)

SUPPLEMENTARY FIGURE 1

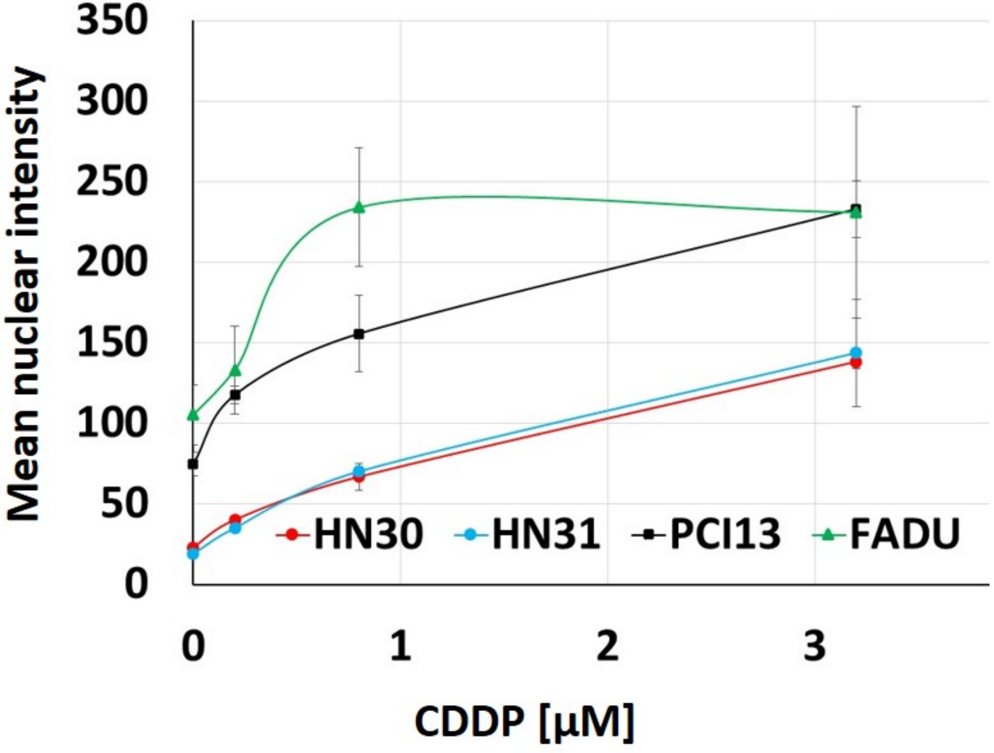

SUPPLEMENTARY FIGURE 2

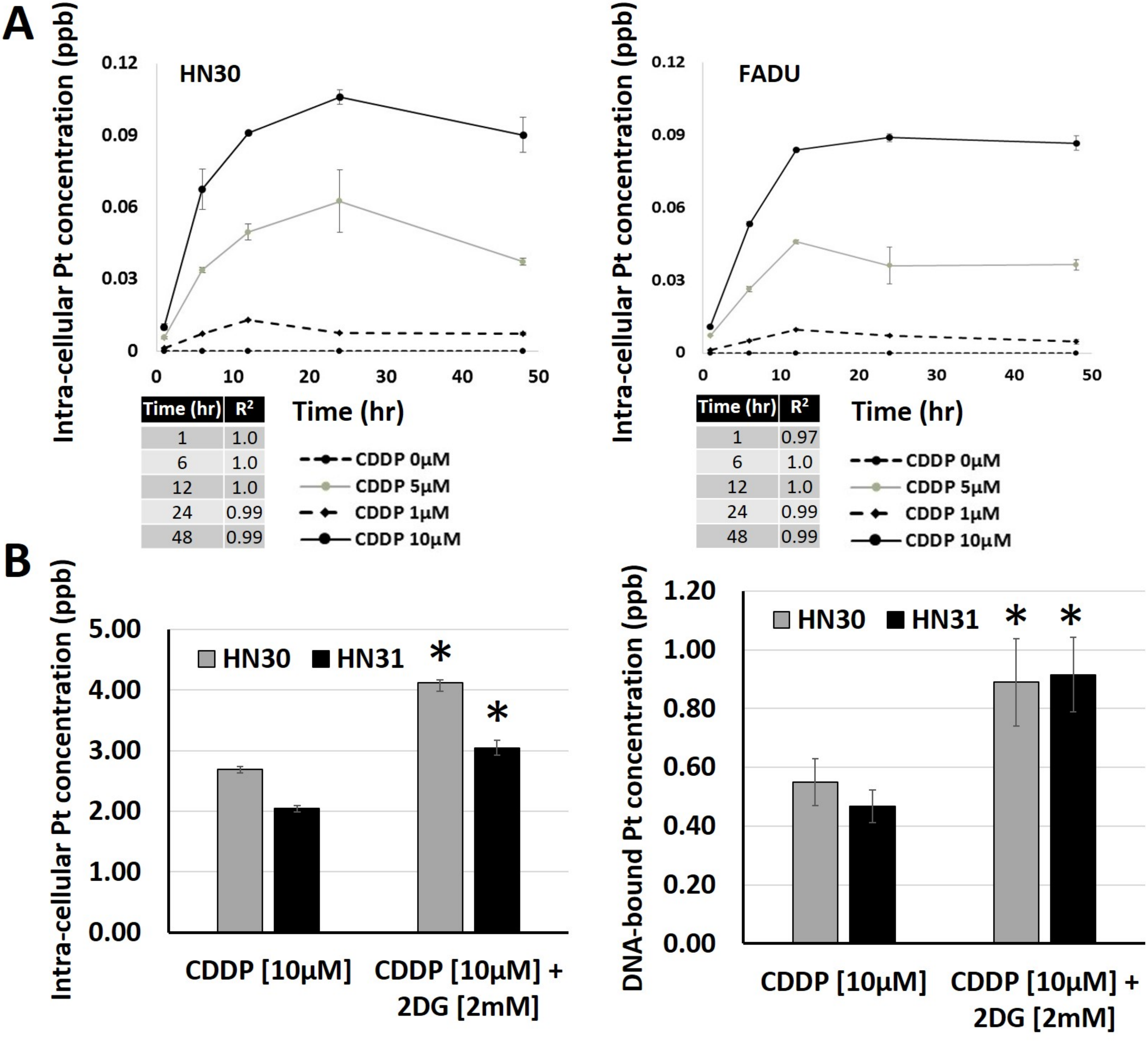

Time (hr)	R^2
1	1.0
6	1.0
12	1.0
24	0.99
48	0.99

Time (hr)	R^2
1	0.97
6	1.0
12	1.0
24	0.99
48	0.99

SUPPLEMENTARY FIGURE 3

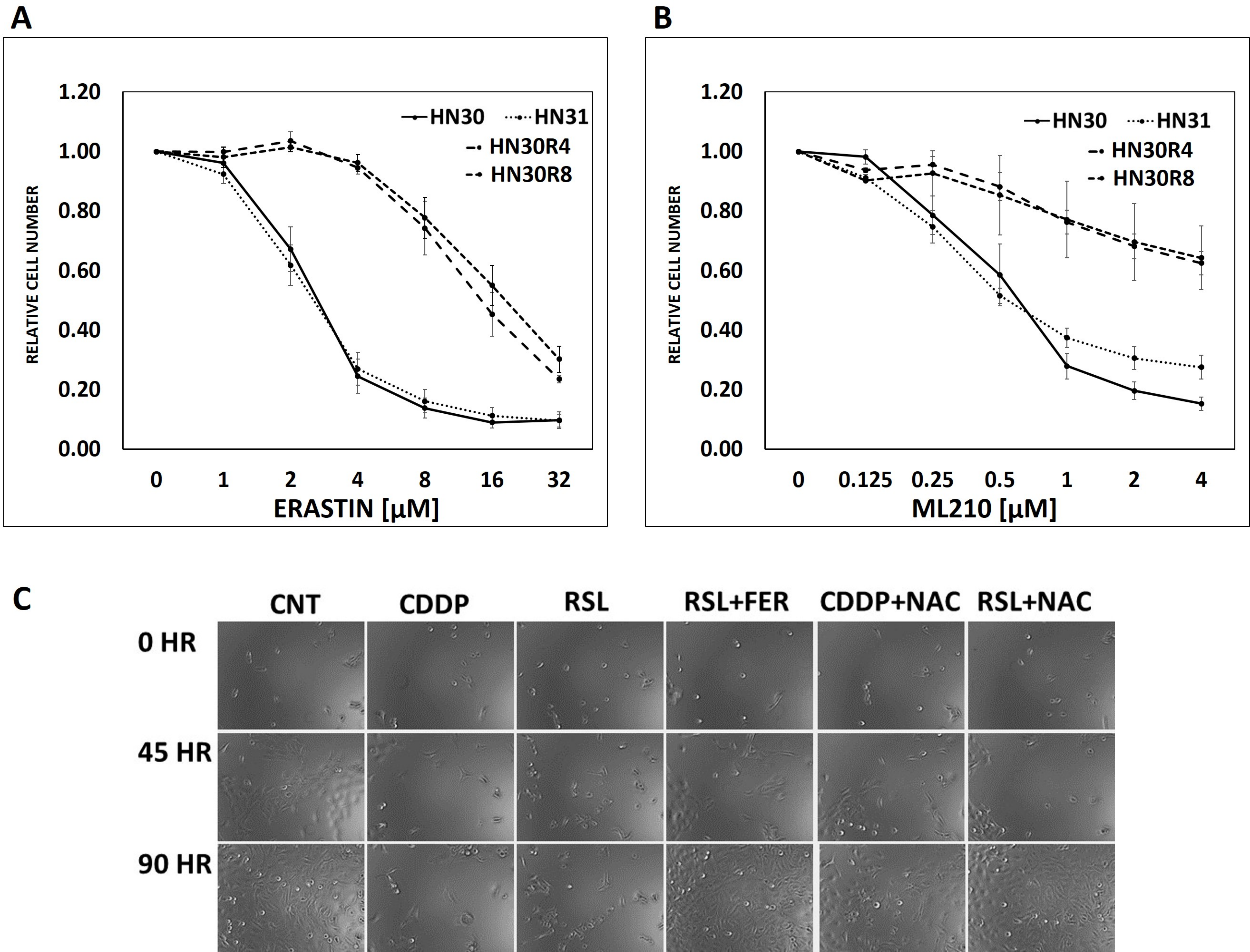

SUPPLEMENTARY FIGURE 4

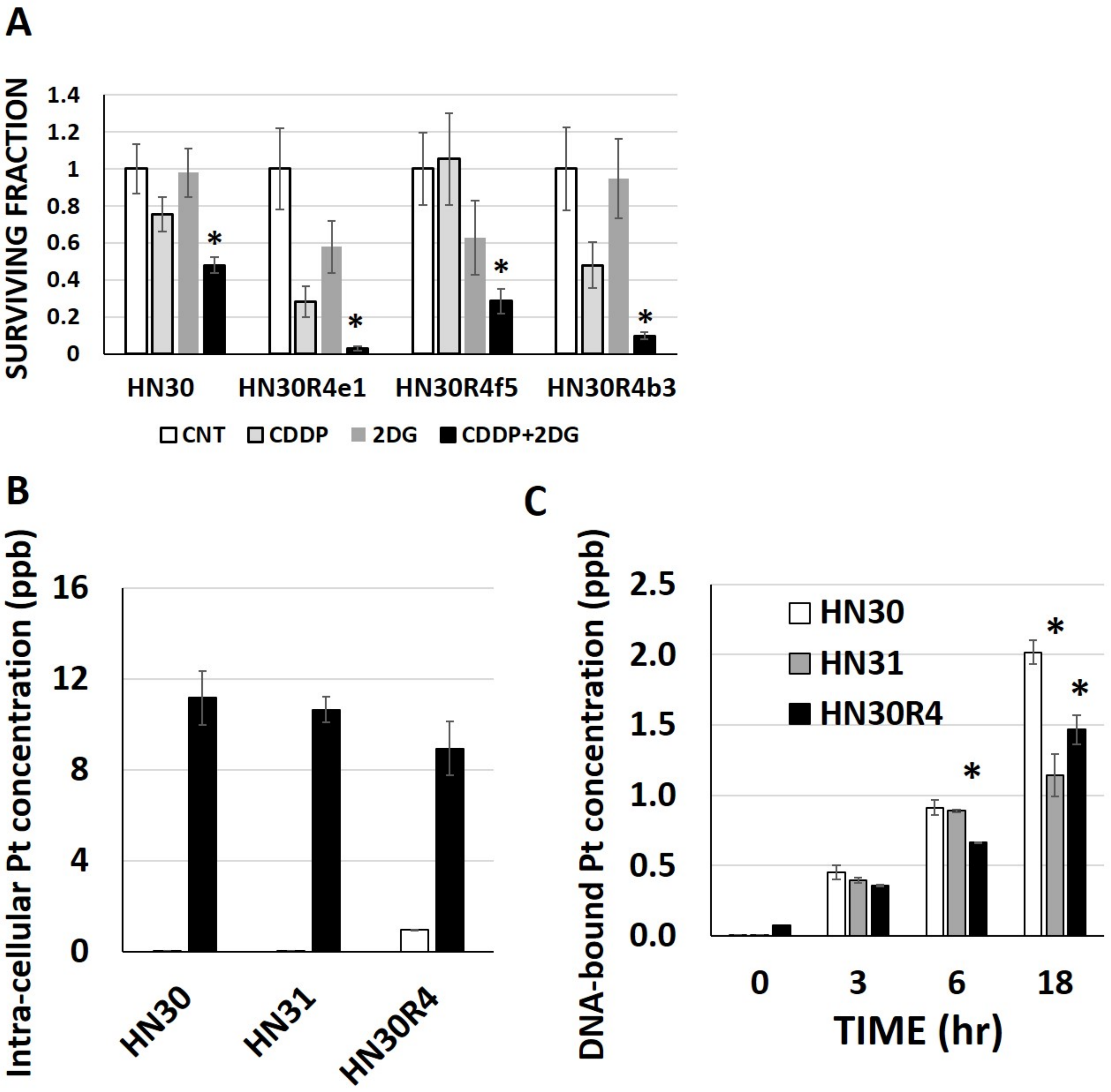

SUPPLEMENTARY FIGURE 5

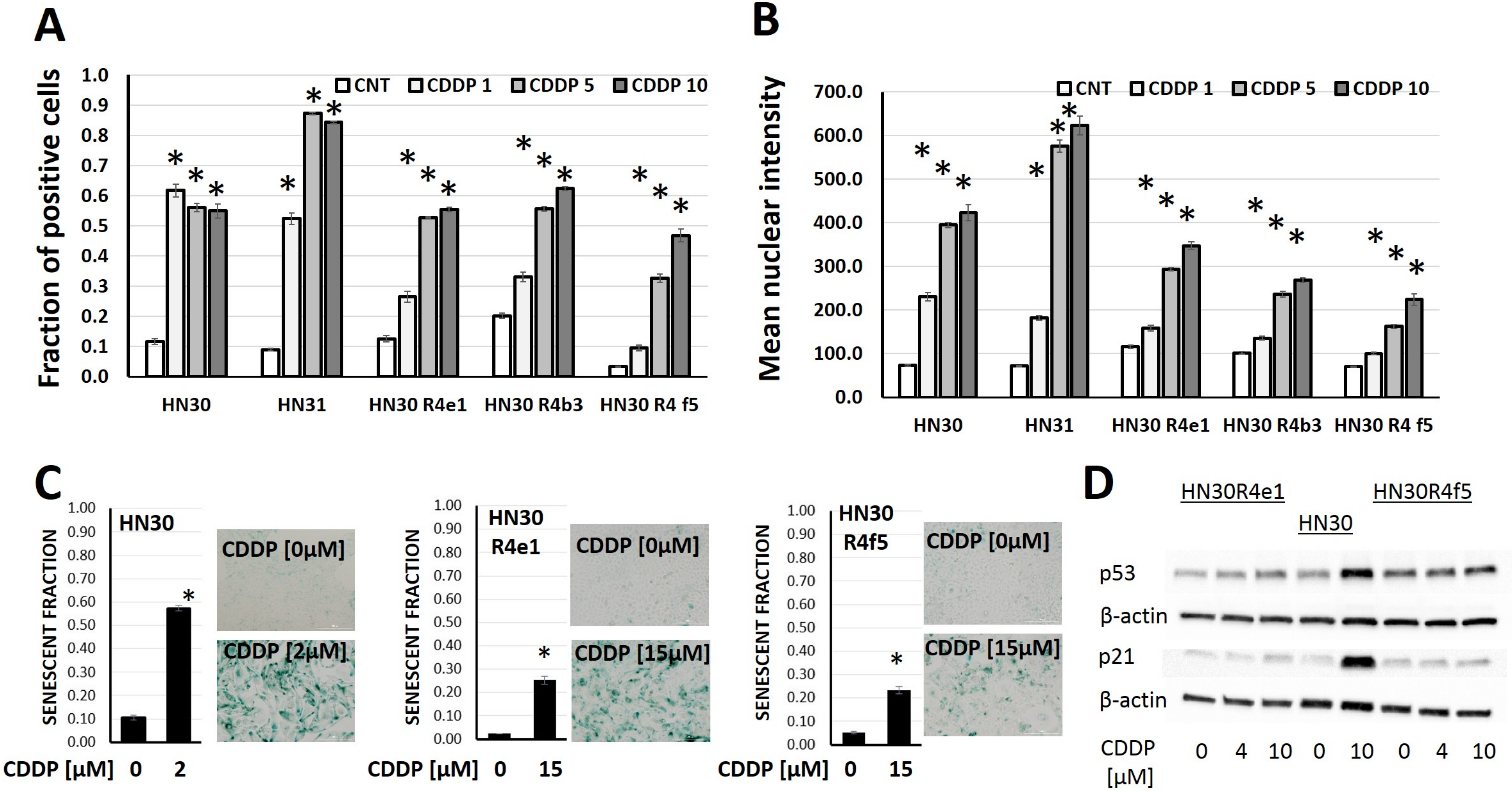

SUPPLEMENTARY FIGURE 6

A

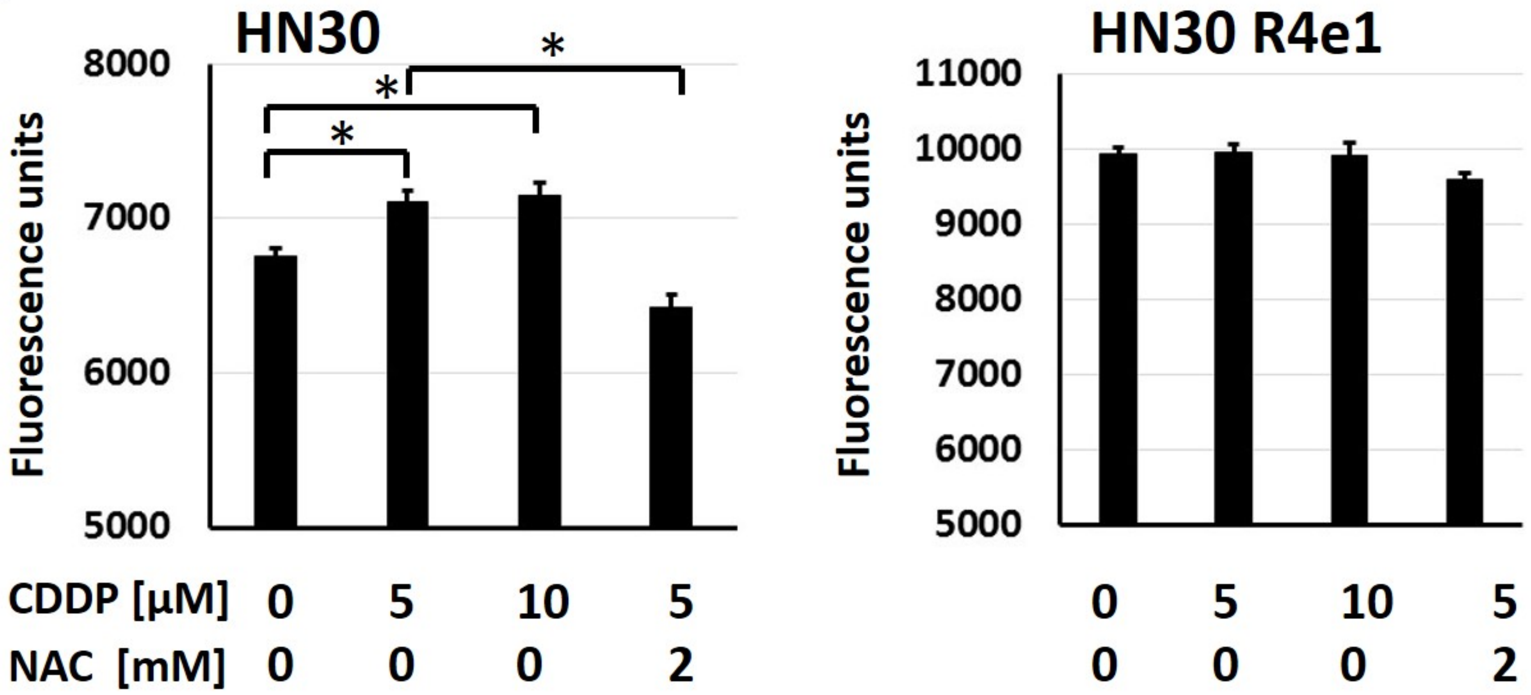

B

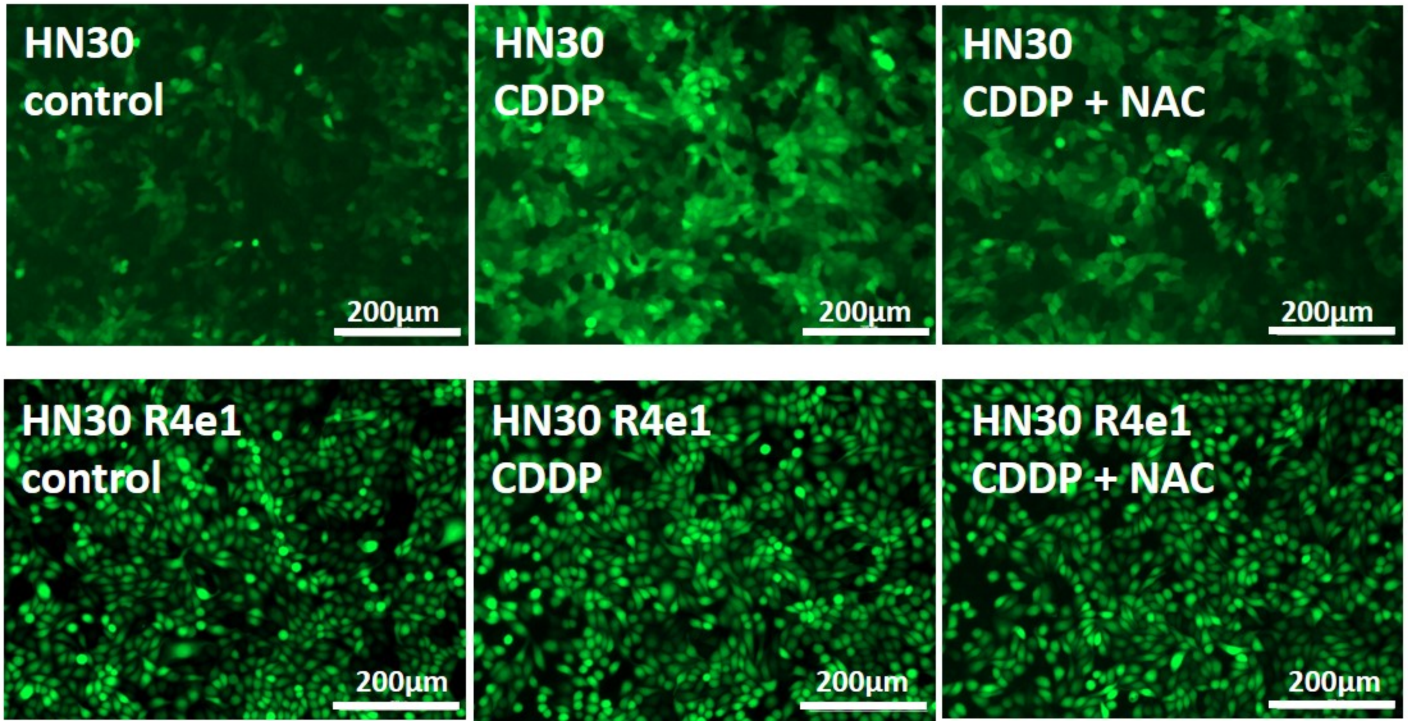

SUPPLEMENTARY FIGURE 5D (complete blots)

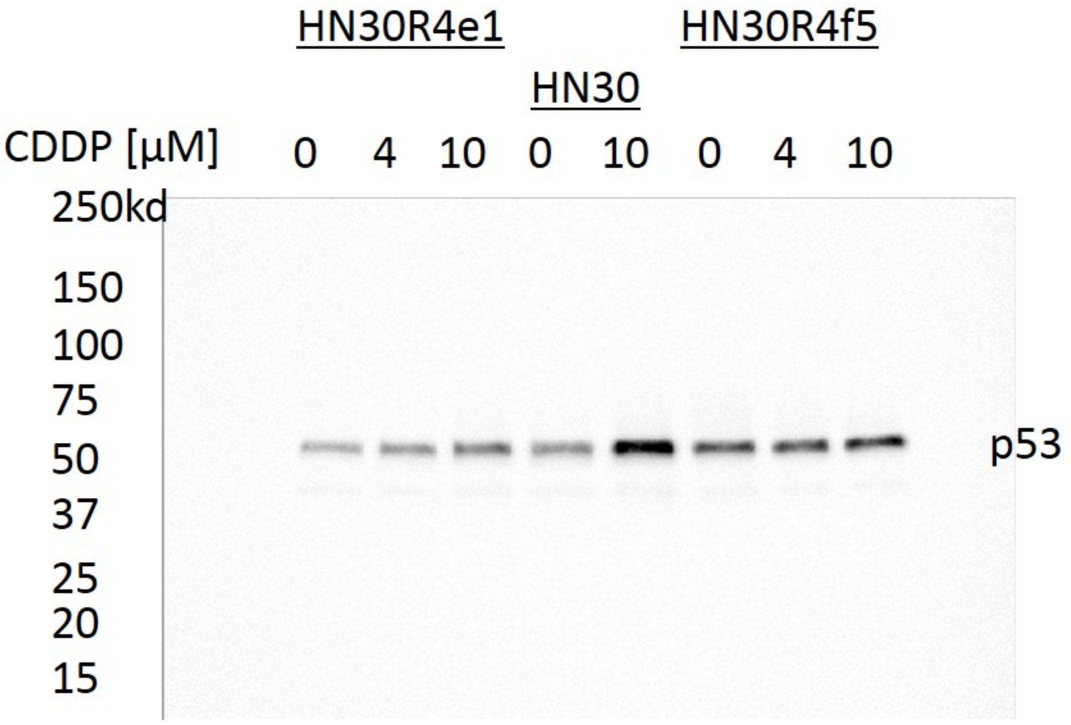

DENSITOMETRY VALUES

4830 8440 21800 10500
5700 7680 11600 12300

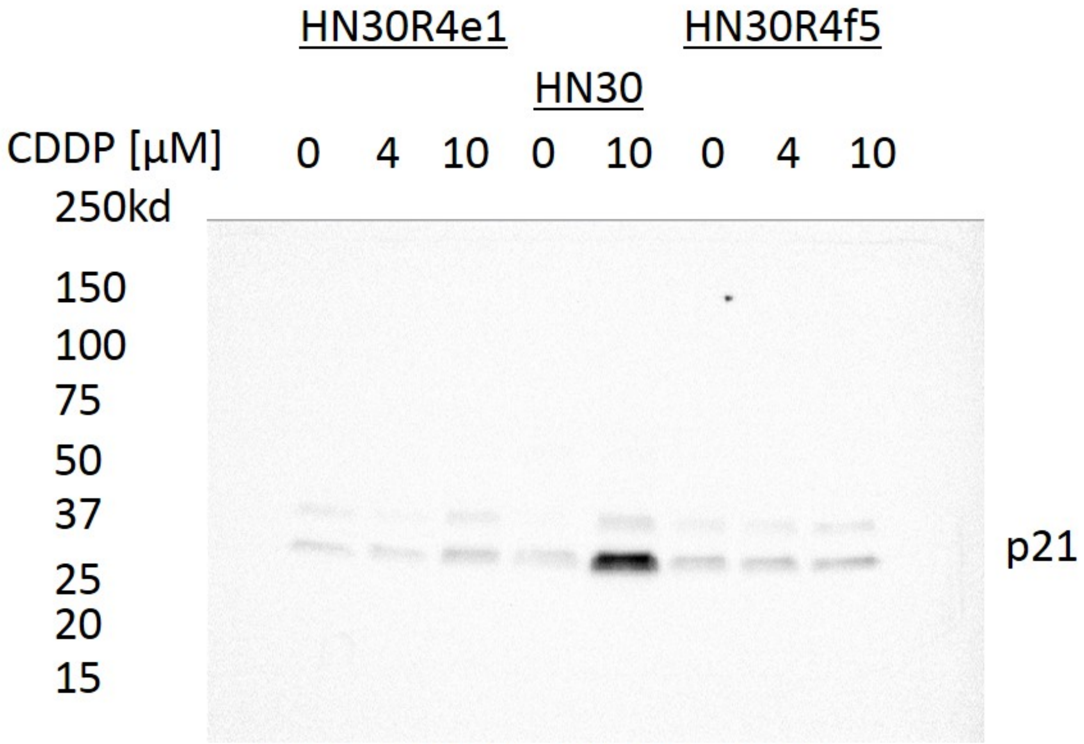

DENSITOMETRY VALUES

8880 17300 100000 15300
7310 12300 18900 20500

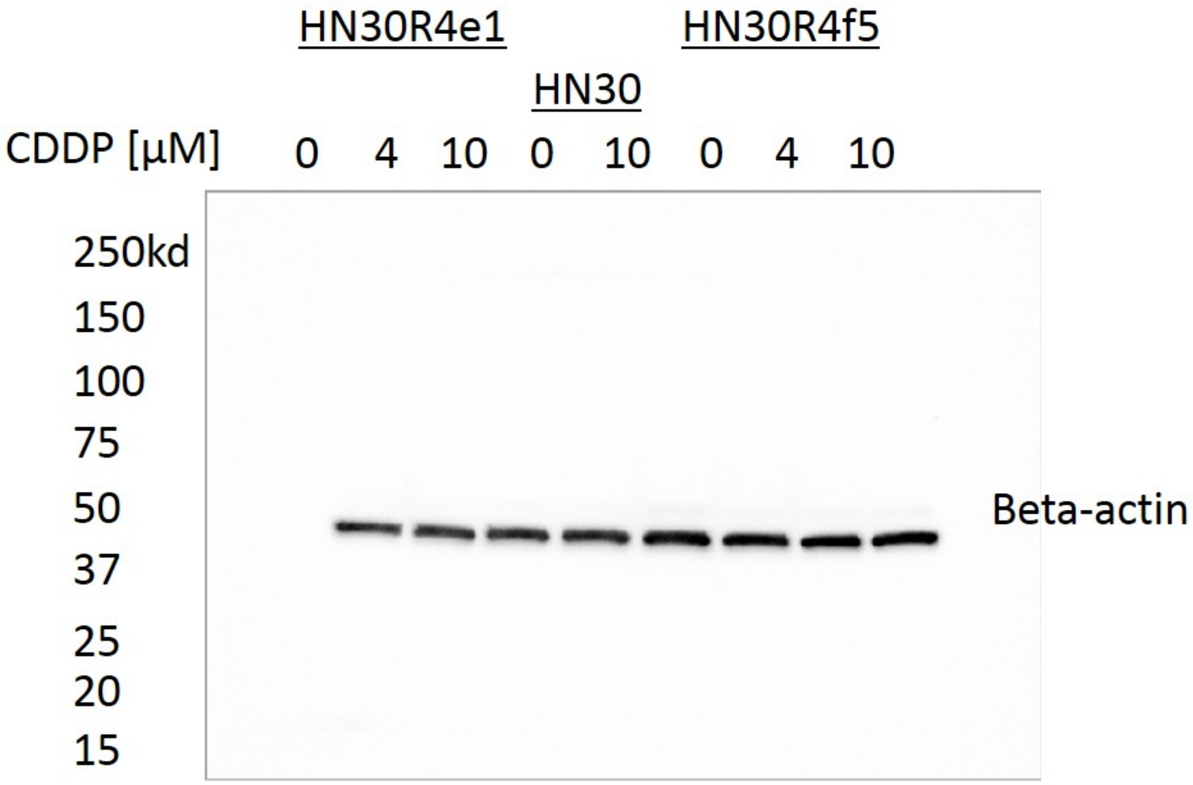

DENSITOMETRY VALUES

87500 92700 100000 100000
85900 100000 100000 100000

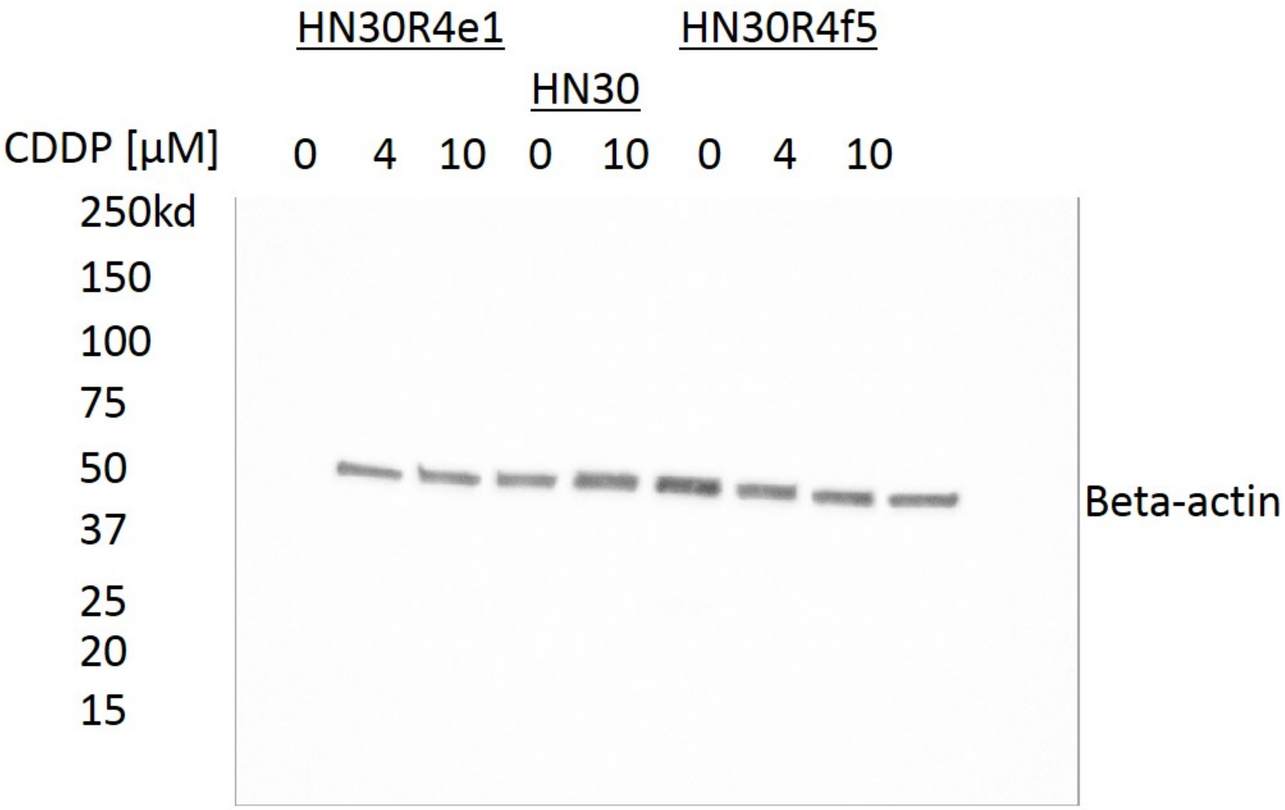

DENSITOMETRY VALUES

46400 48900 88300 58700
47600 71300 59000 57500
